# (Not) "Born in the USA": foreign medical school graduates in the American healthcare system

**DOI:** 10.1590/S1516-31802005000300014

**Published:** 2005-05-02

One in five physicians currently practicing medicine in the United States received their initial medical training in a foreign country.^1^ Nearly 25% of physicians on US postgraduate training programs in 2002-2003 were foreign medical school graduates (FMSGs).^2^ Each year, many residency positions in pediatrics, anesthesiology, psychiatry and family medicine in the United States remain vacant or are filled by foreign-born/foreign-trained physicians.^3^ While possibly refl ecting an overabundance of such residency positions, this situation may also suggest a lack of US medical student interest in these specialties.

While there seems to be no general consensus about physician supply/demand ratio in the United States, and the impact of foreign-trained physicians on health care delivery in U.S. hospitals is still poorly defined, the steadily increasing participation of FMSGs in the American health care system has recently become a politically charged and sensitive issue.^4^ First, it has been reported that almost all states in the United States require that FMSGs pass additional licensure examination(s) and complete more years of postgraduate medical training than their US-born/US-trained counterparts. Second, growing evidence of discrimination against FMSGs in resident recruitment, licensing requirements and unfairly biased perceptions of discipline strength/weakness based on the number of enrolled FMSGs in the residency programs has been collected from several specialties, including psychiatry and anesthesiology.^5-7^

Balon et al.^5^ sought to determine whether there is a selection bias against the applicants who are FMSGs, for US residency training positions in psychiatry. Identical requests for a program application form were sent to 193 residency training programs by two resident applicants, one FMSG and one graduate of a US medical school (USMSG), and the rate and character of the responses were analyzed. The response rate to requests for an application form was significantly higher for the USMSG (159 responses) than the FMSG (87 responses). The quality of responses was also different in several cases. Examples of qualitatively different responses to the FMSGs included statements such as "We do not take international medical graduates"; requests for further information or credentials to be reviewed, following which an application form would be sent if the review were satisfactory; requirement of an unrestricted license in another state; a list of various requirements, such as knowledge of American culture; requests for a curriculum vitae; and requirement of a US medical license examination score of a minimum of the upper 80s. Examples of qualitatively different responses to the USMSGs included a letter from a program director asking the candidate to call with any questions; handwritten notes from a program director; various materials about a program and the city; a handwritten note from a senior or chief resident; a follow-up letter from a chairman asking the USMSG to submit the application; a second application form; and a videotape of a program. The authors concluded that some residency programs in psychiatry were attempting to limit the influx of FMSG applicants at the very first (entry) level: the request for a residency application form. The reasons for this practice were not known, but discrimination based on the applicant's name and the medical school from which she/he graduated could not be ruled out as a possible explanation.^5^

In an article entitled *Are "international" medical graduates second-class anesthesiologists?*^7^ a French-born/trained anesthesiologist wrote: "I do not think that the education I received in a French medical school is in any way inferior to the one that students get in this country (USA)." An FMSG himself, the author of the present letter, whose pursuit of his American (medical) dream has taken him from his native Poland to American medical "heaven", cannot agree more with this statement/conclusion. Having faced many questions about the role/situation/position of foreign- born/foreign-trained physicians in American medicine from many young FMSGs (or FMSGs to be) eager to taste the "American (medical) pie", while lecturing at international anesthesiology meetings around the world, the author of this letter wishes to leave the reader with the following two comments. First, on a personal note, the first tune this author heard upon his arrival at the JF Kennedy Airport in New York, USA was titled *Born in the USA* (by Bruce Springsteen), hence part of the title of this communication *(Not) "Born in the USA"* (and proud of it). Second, on a professional note, it appears to me that the time has come to put to rest the issue of quality/equality of medical education (e.g. international medical school graduates are not as good physicians as their American counterparts^7^) in this "melting pot" country of ours (USA) whose success in great part stems from ethnic and cultural diversity, which obviously needs FMSGs and in which discrimination is illegal.
